# Influence of early life adversity and breed on aggression and fear in dogs

**DOI:** 10.1038/s41598-025-18226-0

**Published:** 2025-10-02

**Authors:** Julia Espinosa, Isain Zapata, Carlos E. Alvarez, James A. Serpell, Anna V. Kukekova, Erin E. Hecht

**Affiliations:** 1 Department of Human Evolutionary Biology, Harvard University, 11 Divinity Ave, Cambridge, MA 02138 USA; 2 Department of Biomedical Sciences, Rocky Vista University, 8401 South Chambers Road, Englewood, CO 80112 USA; 3 Departments of Pediatrics and Veterinary Clinical Sciences, The Ohio State University Colleges of Medicine and Veterinary Medicine, 1900 Coffey Road, Columbus, OH 43210 USA; 4 School of Veterinary Medicine, University of Pennsylvania, 3800 Spruce Street, Philadelphia, PA 19104 USA; 5 Department of Animal Sciences, The University of Illinois at Urbana-Champaign, 1201 Gregory Drive, Urbana, IL 61801 USA

**Keywords:** Psychological stress, Domestic dog, Gene-environment interactions, Psychological wellbeing, Behavioral development, Genetics, Psychology

## Abstract

Among the animals on this planet, dogs are uniquely adapted for life with humans, a status that exposes them to risks of human-mediated traumatic experiences. At the same time, some lineages of dogs have undergone artificial selection for behavioral phenotypes that might increase risk or resilience to stress exposure, providing an opportunity to examine interactions between heritable and acquired traits. In a large-scale study (*N* = 4,497), English-speaking dog guardians reported on their dogs’ life histories, current living environments, and provided observer ratings of dog behavior using the Canine Behavior Assessment and Research Questionnaire (C-BARQ). Our analysis revealed that adverse experiences in the first six months of life, such as abuse and relinquishment, were significantly associated with increased aggression and fearfulness in adulthood, even when accounting for factors such as acquisition source, sex, and neuter status. Additionally, effects of adversity on fearful and aggressive behavior systematically varied at the breed level, suggesting heritable factors for risk and resilience for developing particular phenotypes. Our findings establish that breed ancestry and individual experience interact to show fear and aggressive behavior in pet dogs, confirming that socioemotional behavior is shaped by gene-environment interactions.

**Supplementary Information:**

The online version contains supplementary material available at 10.1038/s41598-025-18226-0.

## Introduction

Dogs (*Canis familiaris*) were the first species domesticated by humans, and their behavioral, physiological, neural, and anatomical phenotypes have been uniquely shaped for, and by, life with our own species^1–3^. This offers an opportunity to study behavioral mechanisms that allow dogs to be well-adapted for their niche within the human sphere. This includes not only the “sunny side” of life with humans, like adaptations for increased sociality^4^ and interspecies communication^5,6^, but also a “dark side” as well. Because they are embedded in human households, family pets are exposed to risks for physical and psychological harm associated with the human world. Furthermore, among extant free-ranging dogs, the leading cause of early-life mortality appears to be human action^7^, suggesting that adaptation for these risks has been part of domestic dogs’ evolutionary history from the beginning.

At the same time, understanding dogs’ responses to trauma exposure has real-world implications for both canine and human wellbeing. Problematic behaviors in dogs, particularly fear and aggression, are leading contributors for relinquishment, bite injuries, and euthanasia^8–10^, posing serious public health and animal welfare challenges. These issues incur substantial societal costs, including medical expenses from dog bites, shelter expenditures for managing displaced animals, and lost productivity due to injury-related absenteeism and legal actions. The high prevalence of these issues, exacerbated by the COVID-19 pandemic, underscores the need to understand the underlying factors driving these behaviors^11–15^. Identifying risk factors for severe fearful and aggressive behavior is essential to explain this trend and guide effective mitigation strategies.

One set of candidate risk factors involves adverse experiences during early development, which can have profound and lasting effects on behavior and health. In humans, childhood maltreatment, resource scarcity, and trauma reliably predict later life psychopathology, including anxiety, depression, and antisocial behavior^16–19^. Primary caregivers also play a crucial role in normative development, with early interactions and ties between child and caregiver laying the groundwork for adult relationships^20^. Similar long-term impacts have been observed in laboratory animal models such as rodents and primates^21–23^. Individuals exposed to resource scarcity or maternal loss under laboratory conditions are more likely to behave anxiously, impulsively, or aggressively^24^. However, the real-world impacts of commonly occurring adverse events remain poorly understood, especially in species that share the human environment as dogs do^25–27^. Understanding these effects in companion dogs is crucial, as they often encounter unique stressors that may predispose them to problematic behaviors.

To address this gap, in a large-scale study (*N =* 4,497) we examined the relationship between various common early life adversities and observer-rated fearful or aggressive behavior in adult dogs. Previous research has largely focused on extreme cases of maltreatment^28–31^ or specific populations like working and service dogs^32,33^, and outcomes of early life experiences in the general pet dog population are underexplored. In rescue dogs, severe physical abuse has been linked with increased fear and aggression towards strangers (both human and canine), while hoarding has been linked with elevated fear and altered social attachment behavior. These studies are compelling evidence for the impact of experiences, while not specifically pointing to the role of timing. In military and service dog populations (often single breed dogs such as German Shepherds or Labrador Retrievers), the first year of life has been identified as a critical time for personality development, with maternal style, puppy raiser experience, and household composition influencing temperament and future success as adults in working dog programs^32–34^. Taken together, these findings suggest that adverse events, particularly during the first year, carry long-term consequences for dogs and so we explored experiences that could occur in the lifetime of a typical pet such as injury, rehoming, and physical or emotional abuse. We hypothesized that dogs exposed to these types of adversities, particularly during the first six months of life, would exhibit higher levels of fear and aggression compared to those without such experiences. Identifying these specific risk factors will provide a foundation for developing targeted behavioral interventions to improve dog welfare and reduce the societal burden of canine behavioral issues.

Consistent with our predictions, results showed that dogs with a history of early adversity showed higher fear and aggression, with life history explaining at least as much variance in each behavior type as sex or neuter status. The adversity-aggression association was most pronounced for events occurring in the first six months of life, aligning with sensitive periods of behavioral development^35,36^. Furthermore, some breeds were at greater risk to the effects of adversity than others, suggesting an important genetic factor in fear and aggressive behavior.

Our results provide novel evidence that common adverse events can have lasting negative impacts on canine behavior in a large community sample of household companion dogs, specifically within a critical risk window in the first six months. Given the high prevalence of childhood adversity^37^ and anxiety-related disorders^38,39^, our results in dogs also inform the etiology of behavioral problems in humans. More broadly, this work underscores the importance of early experiences in shaping lifelong phenotypes and demonstrates the value of companion dogs as a window on the complex interplay of genes and environment in behavioral development.

## Results

**Fig. 1:**
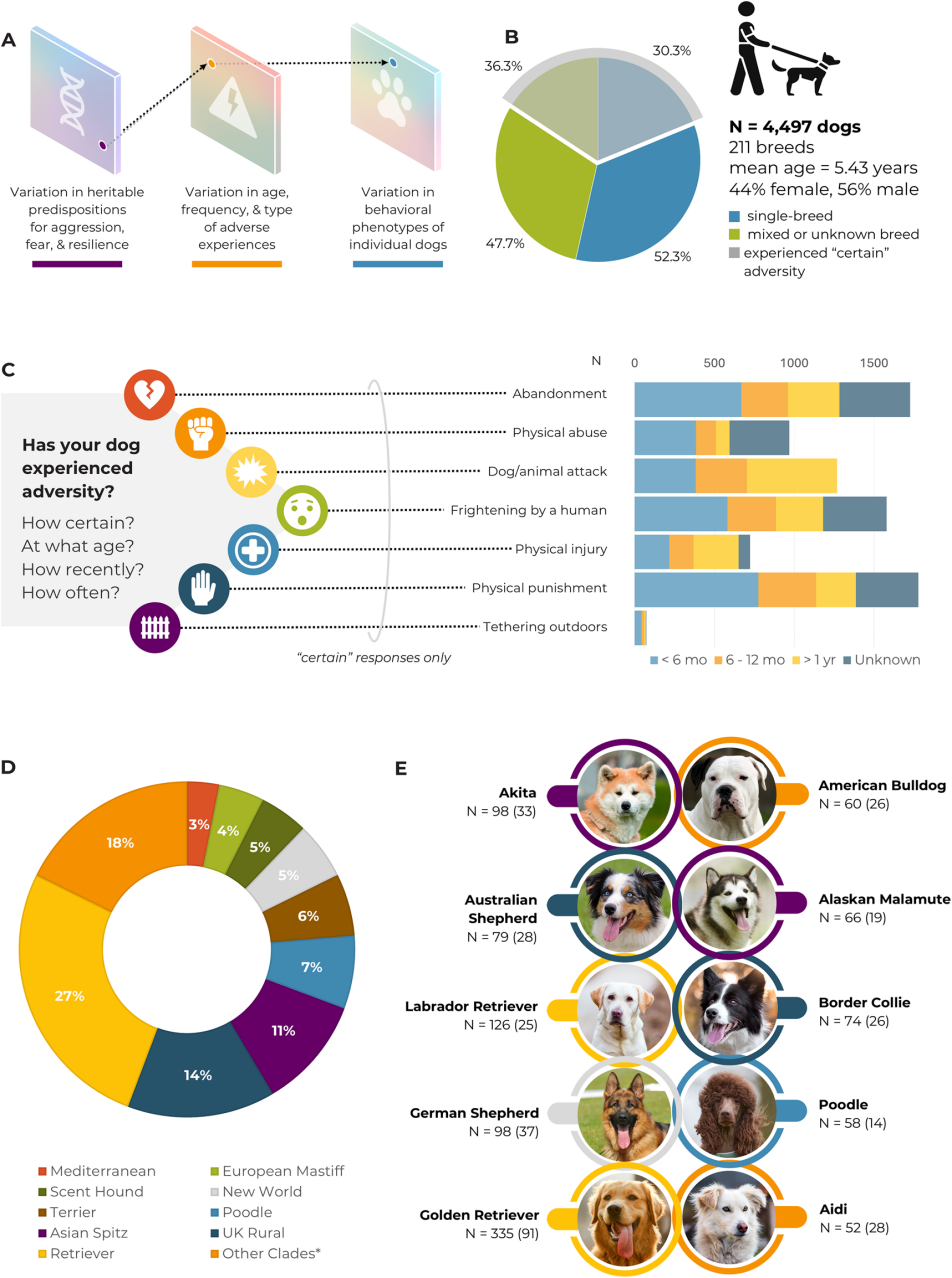
Key information about the included sample of dogs. **A**) Theoretical model including key factors in shaping aggressive and fearful phenotypes in dogs. **B**) Key demographic characteristics of dogs in the study sample. 33% of the entire sample reported certain early life adversity. **C**) Adverse events for which guardians reported their dogs’ life history. The bar graph indicates how many dogs (X axis) were reported in each category (Y axis) by age (fill) at first experience of the event. **D**) Distribution of single breed dogs (*n* = 2,376) across phylogenetic clades with a combined category, “Other Clades*”, for grouping clades with < 3% of the sample (i.e., Alpine, American Terrier, American Toy, Asian Toy, Continental Herder, Drover, Hungarian, Nordic Spitz, Pinscher, Pointer setter, Schnauzer, Small Spitz, Spaniel, Toy Spitz) and single breeds not linked with clades. **E**) Ten most numerous breeds in the sample based on breed n, with early life adversity individuals shown as a subset in parentheses and background colors indicating clade membership.

We compiled usable responses from 4,497 dogs (see Methods). Information about the dogs in this sample is provided Supporting Information Table S2-S3 and in Fig. 1.

### Impact of early life adversity on perceived aggression and fear in dogs

**Fig. 2:**
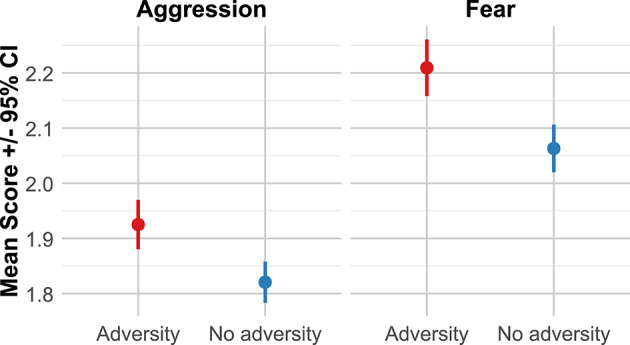
Average perceived aggression and fear scores by adversity.

As preregistered, we examined the association between early life adversity and guardian-perceived aggression and fear in dogs using linear regression models. Our analysis revealed that dogs who experienced adversity within the first six months of life exhibited significantly higher levels of aggression (*M* = 1.93, 95% CI [1.88, 1.97]) compared to those without such a history (*M* = 1.82, 95% CI [1.78, 1.86]; Fig. 2), *t*(4447) = 5.15, *p* <.001. The effect size of early life adversity on aggression was substantial, surpassing the impact of cohabitation with other dogs and sex status, and was comparable to the effects of sex and age (Table 1).

A similar pattern emerged for fear behavior. Dogs who experienced adversity within the first six months of life showed significantly higher levels of fear (*M* = 2.21, 95% CI [2.16, 2.26]) compared to dogs without early life adversity (*M* = 2.06, 95% CI [2.02, 2.11]; Fig. 2), *t*(4447) = 6.45, *p* <.001. The impact of early life adversity on fear was comparable to the effect of cohabitation with children and exceeded the combined effects of individual characteristics such as sex, desexed status, and age (Table 1). Supporting analyses of adversity on subtypes of both aggression and fear revealed a systematic positive effect across all C-BARQ subscales, 0.038 < β < 0.118, except for “*unfamiliar dog aggression”*, β = 0.015, *p* =.292. See supplemental material for further results, and a variation of the main model using *Fédération Cynologique Internationale* breed classification to include more breeds in the analysis^40^ (Table S1).

**Table 1:** Contribution of heritable and environmental factors on perceived aggression and fear.

	Aggression				Fear		
Covariate	*F*		*p-value*	*r*	*F*	*p-value*	*r*
Clade	5.50		< 0.001	0.163	3.87	< 0.001	0.137
Weight (kg)	57.10		< 0.001	0.113	79.10	< 0.001	0.132
Sex	40.16		< 0.001	0.074	0.27	0.602	0.008
Age (years)	18.48		< 0.001	0.074	0.70	0.403	0.025
Desexed (yes/no)	1.50		0.221	0.018	5.88	0.015	0.036
Cohabit with child (yes/no)	152.71		< 0.001	0.185	30.99	< 0.001	0.087
Source of acquisition (breeder/not-breeder)	75.89		< 0.001	0.129	78.12	< 0.001	0.132
Early adversity (yes/no)	26.50		< 0.001	0.078	41.54	< 0.001	0.096
Cohabit with dog (yes/no)	10.53		0.001	0.057	0.01	0.929	0.020
Exercise (hrs)	3.32		0.068	0.025	7.05	0.008	0.039

Note. Estimates of effects obtained from *car* package *(*Version 3.1-2; *Fox & Weisberg*,* 2019).* Numerator *df* is 22 for clade and 1 for all other covariates. *F =* test statistic of Type II Anova. *r* = effect size obtained by taking the square root from the partial *R*^2^ estimates of each covariate from the *jtools* package (Version 2.2.2; *Long*,* 2022*).

### Association between age at time of adversity and perceived fear and aggression

**Fig. 3:**
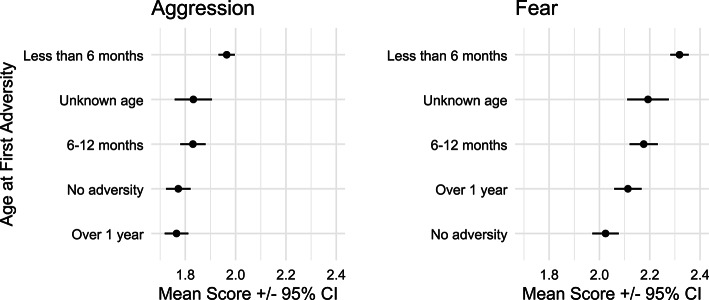
Mean behavior score by age group at time of adversity.

To further investigate the impact of early life adversity on behavior, we compared dogs who were reported as having their first exposure in early life to those who had been exposed in adolescence or adulthood. This analysis revealed a significant main effect of early life adversity on aggression, *F*(4, 4491) = 16.55, *p* <.001. As Fig. 3 shows, dogs that experienced adversity before six months of age were perceived as significantly more aggressive compared to dogs of other age groups; pairwise comparisons between dogs of less than six months and dogs of all other age groups yielded t-values ranging from 3.23 to 6.51, and p-values ranging from .001 to .011. Additionally, dogs that had experienced a greater number of adverse events were perceived as more aggressive, *F*(1, 4491) = 18.70, *p* <.001. Results were similar for fear behavior, main effect: *F*(4, 4491) = 21.24, *p* <.001, and pairwise comparisons between dogs of less than six months and other age groups yielded t-values ranging from 2.73 to 8.42, and p-values ranging from .001 to .051, with dogs experiencing a greater number of adverse events perceived as more fearful *F*(1, 4491) = 18.94, *p* <.001.

### Impact of adversity on perceived aggression and fear by individual breeds

**Fig. 4:**
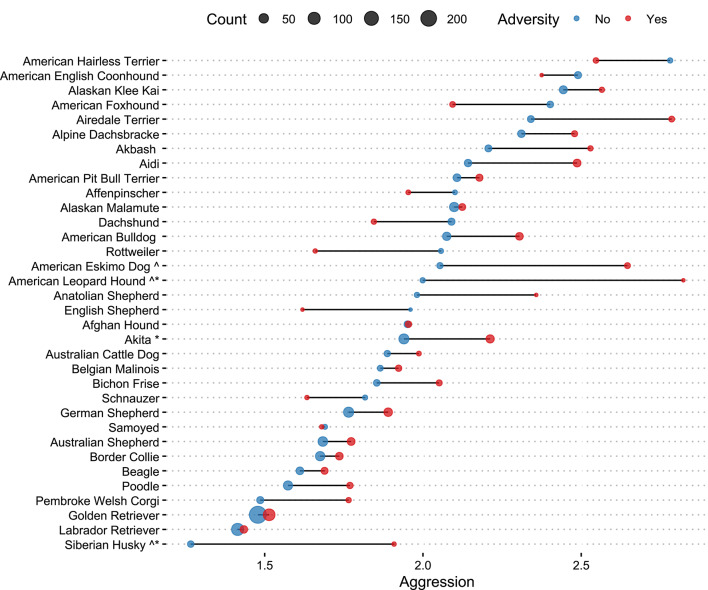
Average aggression by breed and history of adversity. *Note*. Estimated means of average aggression for dogs with and without history of adverse life experiences by breed, obtained from a linear model with adversity, breed, and adversity x breed interaction. We focused on breeds with at least five individuals per condition. To account for unequal sample sizes by breed, we performed a Monte Carlo permutation test (*k* = 5000) —a non-parametric procedure testing the null hypothesis by generating a distribution of permuted data under the assumption that the observed effect is due to chance. Carats next to the breeds indicate significant difference at *p* <.05 in linear regression, while stars next to the breed reflects proportion of permuted statistics that were as extreme or more extreme than the observed statistic (also *p* <.05). Dot size corresponds to the sample size within experience level for each breed. In no breeds did early life adversity cause a significant reduction in aggression scores. Breeds which seem to indicate this pattern have small sample sizes and large error estimates.

To explore the impact of selective breeding on reports of aggressive and fearful behavior, in a follow-up analysis we examined individual breeds. We fitted a linear model that included breed, adversity, and a breed x adversity interaction for breeds represented by at least 5 dogs with and 5 dogs without early life adversity (*k* = 34; *min n*/cell = 5). As Fig. 4 shows, some breeds showed a significantly greater difference in mean fear and aggression between individuals with and without adversity, including American Eskimo Dogs, *t*(1658) = 2.31, *p* <.021, American Leopard Hounds, *t*(1658) = 2.26, *p* =.024, and Siberian Huskies, *t*(1658) = 2.05, *p* =.040. Conversely, some breeds showed very little difference in aggressive behavior after early life adversity, such as Golden Retrievers and Labrador Retrievers, 0.135 < |*t*s*| ≤* 0.45, *p*s > 0.685 (Fig. 4). Parallel analyses with a non-parametric Monte Carlo permutation test confirmed these results (Supporting analyses with a more conservative threshold, min *n/*cell = 10, yield very similar overall results. Figure S1-S2).

**Fig. 5:**
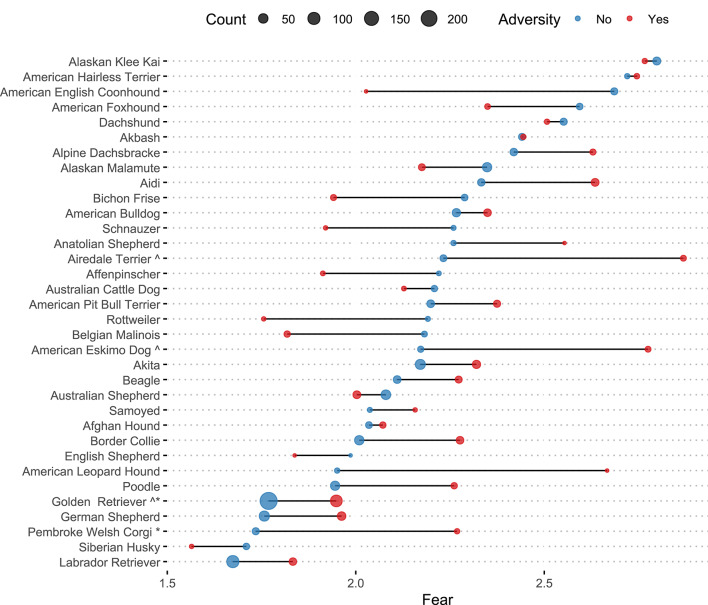
Average fear by breed and history of adversity. *Note*. Estimated means of average fear for dogs with and without history of adverse life experiences by breed, obtained from a linear model with adversity, breed, and adversity x breed interaction. We focused on breeds with at least five individuals per condition. To account for unequal sample sizes by breed, we performed a Monte Carlo permutation test (*k* = 5000) —a non-parametric procedure testing the null hypothesis by generating a distribution of permuted data under the assumption that the observed effect is due to chance. Carats next to the breeds indicate significant difference at *p* <.05 in linear regression, while stars next to the breed reflects proportion of permuted statistics that were as extreme or more extreme than the observed statistic (also *p* <.05). Dot size corresponds to the sample size within experience level for each breed. In no breeds did early life adversity cause a significant reduction in fear scores. Breeds which seem to indicate this pattern have small sample sizes and large error estimates.

Applying an analogous set of tests to the fear data, Airedale Terriers stood out as having a stronger association between adversity and increased fear, *t*(1658) = 2.41, *p* =.016, as did American Eskimo Dogs, *t*(1658) = 2.14, *p* =.033, and Golden Retrievers, *t*(1658) = 2.04, *p* =.042, while other breeds showed non-significant trends in the same direction, Pembroke Welsh Corgis, *t*(1658) = 1.87, *p* =.062, and American Leopard Hounds, *t*(1658) = 1.79, *p* =.074. Labrador Retrievers again showed very little difference in fear behavior after early life adversity, *t*(1658) = 0.96, *p* =.320 (Fig. 5).

## Discussion

Fearful and aggressive dog behavior are serious issues, impacting public health and wellbeing in dog-human relationships. To better understand factors influencing dog fear and aggression, we examined the effect of adverse experiences on behavior phenotypes with a large survey of English-speaking companion dog guardians. Using guardian self-report data to look at behavior outcomes of adversity, we found that dogs with a known history of adverse experiences in the first six months of life scored higher on scales of fearful and aggressive behavior compared to dogs without a history of adversity, or that experienced adversity in later life (Figs. 2 and 3). There was significant variability across dog breeds (Figs. 4 and 5), with some breeds appearing to have increased risk for developing fearful and aggressive behaviors after early life adversity, particularly breeds historically bred for livestock guarding or bringing down game. Conversely, other breeds seemed relatively unimpacted by early life adversity, suggesting a potential resilience to stress. Overall, these results demonstrate long-term negative impacts of adversity on companion dog behavior and suggest critical gene-environment interactions underlying aggressive and fearful behavior. Though there is much anecdotal evidence about the impact of experiences on dog behavior, there has been very little empirical work addressing this at the general population level. Our study provides a novel vantage point to assess behavioral outcomes of adversity in a large, community sample of companion dogs.

### Impacts of early life adversity on dog behavior parallel those in humans and other mammals

Our results are in line with the findings of prior work with humans, dogs, and other mammals. Early life adversity in humans has received substantial scientific attention, and the links between early life adversity and mental health disorders such as depression and anxiety have been well documented (for reviews see^41–44^. Similarly, in non-human primates, altered emotional reactivity and social behavior have been linked with abusive maternal styles^45^, and in rodents early maternal separation is associated with increased anxiety when exposed to stress in later life^46,47^. Previous canine studies have focused on situations of extreme adversity such dog victims of abuse or neglect, or in special working dog populations, providing strong evidence that traumatic experiences have a negative impact on fear and aggressive behavior^29,48^ and that the first year of life is critical for development^32–34,49^. Here we extended these findings in the general companion dog population, and also identified the sensitive period when dogs are most vulnerable to stress. Combined, this research provides strong evidence that a similar phenomenon occurs in dogs as in other mammals: early life adversity is associated with later increases in fear and aggressive behavior, and the earlier the stimulus is experienced the more pronounced the effect.

### Breed differences in behavior

In the absence of adversity, previous research has documented that there are breed differences in fearful and aggressive behavior linked to genetic predispositions^50–53^. Together with the evidence that environmental factors influence behavior expression^36,49,54,55^, this suggests possible breed differences in behavior may be related, in part, to differences in sensitivity to early life adversity. To assess this possibility, we examined behavior scores of a subset of well-represented breeds in the sample. We compared within-breed behavior differences for dogs with and without early life adversity. This approach identified a few breeds with significant differences in fear and/or aggression as a function of adverse life history: American Leopard Hound, American Eskimo Dog, Siberian Husky, and Golden Retriever. Notably, some breeds show a significant impact of adversity on aggression but not fear, and vice versa. This suggests that some breeds may have a lower threshold to developing problematic behaviors of a particular type, or that there is a heritable risk component that increases susceptibility to stress. Such genetic risks have been found in other species, such as humans^56,57^ and laboratory animal species (e.g., rats^58^; mice^59^; monkeys^60,61^. We also found some breeds that showed an inverse pattern, with exposure to early life adversity associated with reduced fear and aggression (though none of these instances were statistically significant). A similar outcome has been observed in other species exposed to early life stress^62^ and there are hypotheses about the adaptive role that stress may have in boosting resilience and preparing some individuals to cope with challenges in later life (for reviews see^63,64)^. Future research with larger sample sizes per breed is necessary to interpret the behavioral impacts of adversity in these breeds and to more deeply explore breed-level risk or resilience to stress.

### Adversity timing and amount are critical

Dogs undergo rapid physical^65,66^, behavioral^67,68^, and cognitive development^69^ in the first weeks of life, during the so-called “socialization” period (weeks 3–12). Disruptions to these processes through impoverished early rearing environments^36^, inadequate maternal care^70,71^, and inconsistent or overwhelming social interactions^35^ during socialization have been identified as sources of behavioral problems. Our study examined the impact of events during the socialization period and part of the juvenile period (3–6 months), extending past the typical age of interest and capturing events that occur after separation from the dam and leaving the breeder. The results suggest that adverse events throughout this time period are important for behavioral development: compared to the outcomes from adverse experiences at other life stages, dogs that experience adversity in the first 6 months of life show on average higher levels of fearful and aggressive behavior.

### Additional genetic and environmental factors that affect fear and aggression

While adverse experiences during development have a substantial impact in shaping adult behavior, long-term environmental factors and individual differences contribute as well. Our study identified age, sex, and body size (i.e., weight) as key characteristics influencing behavior profiles, consistent with previous findings^51,52,72,73^. Looking beyond biological factors, we also found that environmental factors such as living with children were associated with increased fear and aggression. This is consistent with prior work examining trends in human directed aggression^74,75^, as children are frequent targets of aggression. They are less likely to interpret and respond to dog behavior correctly, and when cohabiting with dogs there are increased opportunities for inappropriate or unsupervised interactions (e.g., taking away toys or food) that can increase risk. A separate reason that dogs cohabiting with children may be scored higher is that parents have a lower sensitivity threshold for interactions involving their child. What might be considered normal boundary setting by a dog when interacting with an adult could be perceived as more threatening and dangerous when involving a child. Future research should investigate this possibility in greater depth.

### Limitations and future directions

We relied on guardians’ reports to obtain a large and diverse group of dogs with varied life experiences—the only pragmatic way to capture the diversity of canine aggression at scale. Because no systematic life history records exist due to a lack of centralized pet databases and registries, the only alternative approach would have been to recruit and track dogs in a prospective longitudinal study. While this approach may be perceived as the golden standard, it is impractical when our goal is to understand the diversity of experiences and the differential impact of adversity across breeds. Also, in the context of adversity-aggression research, prospective longitudinal studies can distort the results due to Hawthorne effects on dog guardians (i.e., guardians would receive more information about the study aims and hypotheses and likely alter their behavior ratings in the wake of an adverse event). Critically, while our approach prevents us from independently verifying life histories, we controlled for the guardian’s level of confidence about adverse events, and in the main analyses only classified early life adversity as such if the reports included concrete information about life history. In this way we controlled for the impact of guardians’ lay theories about the causal nature of adversity on behavior, especially in the case of dogs adopted as adults (e.g., “Charlie is scared of men, probably because a man abused him at some point in his past”). Another common challenge in survey studies is that there is variability in guardians’ knowledge of behavior and ability to report and describe it in the context of their dog. Though C-BARQ has been validated in different populations of dog guardians and in multiple languages and cultures^76,77^ (also see our supplementary analyses on the psychometric structure of C-BARQ), an unavoidable limitation of a survey-based approach is the use of a small number of labels to describe behavior. Consequently, some guardians may have underreported fearful or aggressive behavior in their dog due to limited examples provided. Finally, though we found that subtypes of fearful and aggressive behavior were consistently impacted, it is possible that meaningful heterogeny exists across subtypes of problematic behavior by adversity types. For example, prior work found that resource guarding was not more frequent in dogs that had experienced resource scarcity through criminal cruelty (i.e., hoarding, dog fighting, or puppy mills)^31^, suggesting that food-related aggression may be influenced by factors other than experience, such as heritable traits —an important avenue for future research.

## Conclusions

This study assessed canine behavioral profiles and lifetime stressful experiences using the largest sample of companion dogs yet assembled. We found that stressful events occurring before six months of age are associated with significant increases in undesirable behavior. Furthermore, we observed significant effects of heritable, biological factors like sex and breed ancestry, as well as environmental and experiential factors like presence of children in the household and prior shelter stays. This points toward clear opportunities for future research to examine the genomic, physiological, and neurological mechanisms underlying the effects observed here to facilitate diagnosis, treatment, and prevention of canine fear and aggression problems. Future research in both basic and applied sciences can move toward identifying genetic variants linked to problematic behaviors to inform breeding practices that focus on temperament for particularly at-risk breeds, and support targeted rehabilitation strategies for dogs exposed to early life adversity using medication and behavior modification training. It can also guide rehoming decisions for particularly at-risk dog breeds by matching them with knowledgeable guardians in non-triggering environments. More broadly, our results identify substantial overlap in the adverse life experiences influencing psychological wellbeing in dogs, humans, and other diverse species.

## Materials and methods

### Participants

All materials and procedures were reviewed and approved by the Institutional Review Board of the Harvard University-Area Committee on the Use of Human Subjects and the methods were carried out in accordance with all relevant guidelines and regulations. Participants were recruited through social media (Twitter, Facebook, Instagram, and LinkedIn), with paper fliers, and through in-person outreach at dog sporting and local community events. A sample of adult, English-speaking dog guardians (hereafter guardians) gave informed written consent to participate in the study and subsequently provided information about their dogs’ behavior and life history (*N* = 4,497, *M*_*Age*_ = 5.42 years, *female* = 44%; *spay/neuter status*: intact_Female_ = 302/intact_Male_ = 587, *single breed* = 52.3%). The sample included 211 distinct dog breeds (Fig. 1; Table S2-S3). Most guardians (*n* = 2,499) reported that they were the dog’s first caregiver after separation from the dam. Of these, 1,485 dogs were acquired directly from a breeder. Other sources of acquisition included animal shelters (*n* = 852), foster organizations (*n* = 1,173), family or friends (*n* = 576), and other various sources (*n* = 410; e.g., found the dog while traveling). We excluded additional incomplete responses (i.e., more than half of the aggression scale or fear scale were left blank; *n* = 210), bot-like responses (i.e., strings of nonsensical text in open response fields or identical responses across multiple entries; *n* = 765) and participants with a dog less than 6 months of age (*n* = 101). To incentivize participation, we had a prize drawing for 20 $50 (USD) gift cards. Information about dogs in this sample is provided in Supporting Information (Tables S2-S3) and in Fig. 1.

### Procedure

The study hypotheses and methods were pre-registered on OSF on October 4, 2022. We conducted data collection with an online questionnaire between October 5, 2022 – July 31, 2024. Guardians were asked to provide their dogs’ life and health history, a range of individual characteristics (e.g., the dog’s weight, sex, age, breed), and completed an assessment of their dog’s current behavioral profile (via the Canine Behavior Research Questionnaire, C-BARQ^78^.

### Materials and measures

#### Early life adversity

The life history section included questions about adverse events the dog may have experienced. These adversity questions were based on tools used in child psychiatry, in particular, a parent-report tool developed to describe exposure to adversity for children under seven, the *Traumatic Events Screening Inventory - Parent Report Revised *(TESI-PRR^79^;. We asked guardians about seven distinct types of adversity. To reduce mischaracterization of abstract categories, we provided concrete examples of each adversity type: (i) physical punishment/corrections (e.g., alpha roll, holding the dog’s mouth shut); (ii) separation from or lack of a primary caregiver (i.e., time in a shelter, living on the street); (iii) physical abuse (e.g., hitting, kicking); (iv) being attacked by a dog or other animal; (v) being intensely frightened by a person or in situations involving a person; (vi) suffering a serious physical injury/the threat of a serious physical injury (e.g., being hit by a car, fire, near drowning); and (vii) chaining/tethering outdoors for long periods of time. For each, guardians reported whether their dog had experienced such adversity via two-step chain prompts. First, guardians responded whether a specific adversity type was present, by selecting one of the four response options (“*yes”*,* “I suspect so”*,* “no”*, or “*I don’t know”*). Affirmative responses (“yes” and “*I suspect so*”) triggered concrete follow-up questions that collected information on (to the best of the guardian’s knowledge) the dog’s age at the time of the adverse experience, number of times the same type of experience had occurred over the lifespan, and when the most recent experience of adversity had occurred. We operationalized early life adversity as adverse events occurring before six months of age and created a binary variable to indicate the presence (1) or absence (0) of early life adversity in dogs. Using a conservative approach, in the main analyses we solely focused on non-ambiguous responses, coding strong affirmative responses (“*yes*”) as present for early life adversity. To further reduce ambiguity, in our core analyses we only examined responses that provided concrete details concerning adversity time points in the follow-up questions. Thus, only responses that indicated strong certainty about an adverse event that occurred during the first six months of life would be included in the “early life adversity” group for all analyses. This approach controlled for the possibility that speculative responses about dogs adopted from shelters would be disproportionately impacting results.

#### Perceived aggression and fear

Next, we assessed behavior profiles of dogs using the full 5-point C-BARQ^78,80^, a validated guardian-report behavior assessment tool. To prioritize collection of the subscales most immediately relevant to our hypotheses, guardians first reported on the aggression, fear, and separation-related behaviors prior to sub-scales measuring training, excitability, attachment and attention-seeing, and miscellaneous behavior. Guardians rated their dog’s typical behavioral response on 27 aggression items and 18 fear items (0 = “no visible signs of aggression/fear” to 4 = “serious aggression: snaps, bites, or attempts to bite”/“extreme fear: cowers, retreats, or hides”; see OSF for item wording and scale flow). Reliability of fear and aggression C-BARQ ratings was high (aggression α = 0.94; fear α = 0.90). Therefore, for the main analyses we averaged the respective items of perceived aggression and fear within each scale, with higher scores indicating a more extreme behavioral response. We also performed exploratory analyses to understand the effect of adversity on specific subtypes of aggression and fear^78,80^. For measurement model fit and results for subtype analyses, please see Supporting Information.

#### Breed grouping

To account for heritability in behavior, we categorize breeds according to phylogenetic clades^81^. This approach allowed for a data-driven framework for breed categorization and facilitated analysis and interpretation of the results using the latest in canine phylogenetic analyses. Breed groups include: i- Alpine; ii - American Terrier; iii – American Toy; iv – Asian Spitz; v - Asian Toy; vi - Continental Herder; vii - Drover; viii – European Mastiff; ix - Hungarian; x - Mediterranean; xi – New World; xii – Nordic Spitz; xiii – Pinscher; xiv – Pointer Setter; xv - Poodle; xvi – Retriever; xvii – Scent Hound; xviii – Schnauzer; xix - Small Spitz; xx – Spaniel; xxi – Terrier; xxii – Toy Spitz; xxiii – UK Rural. We categorized breeds that were not included in Parker et al., (2017) as “Non-Clade breeds,” and dogs of multiple breed or uncertain genetic ancestry as “Mixed breed” (see Fig. 1 and Table S2-S3 for detailed demographic information of the sample).

### Analytic procedure

We analysed the data in *R* (Version 4.3.2^82^; data and analysis script are on OSF). To examine our preregistered hypotheses about the associations between early life adversity and perceived aggression and fear, we performed linear regression analyses with aggression/fear as criterion variables, with clade, sex (male/female), spay/neuter status (desexed: yes = 1/no = 0), age (years, continuous), weight (kg, continuous), cohabitation with other dogs or children (yes = 1/no = 0), source of acquisition by the guardian (breeder vs. not-breeder), and dog’s average weekly exercise (hours) as predictors. Another regression model explored whether the association between adversity and perceived aggression varied by the age at which the dog experienced the adversity. To this end, age at time of first-reported adverse event was categorized as early (before 6 months of age), mid (6–12 months of age), or late (1 year or older) and was included in the model with an interaction term quantifying the number of known adversities.

To explore whether the effect of early life adversity varies by specific breeds, we performed a separate regression analysis on breeds that included at least five participants with, and five participants without life history of adversity, respectively (*k* = 34 breeds; 36.7% of the total sample). To confirm that our findings were not driven by outliers, we also performed a non-parametric Monte Carlo permutation test with 5000 permutations. We shuffled the adversity labels within each breed group to calculate permuted differences in mean behavior score for fear and aggression. The observed differences were compared to the permuted distribution to obtain p-values for each breed, thus ensuring that the observed associations were not driven by outliers or specific data distributions.

## Supplementary Information

Below is the link to the electronic supplementary material.

Supplementary Material 1

## Data Availability

Materials, data and analysis scripts are available on OSF (https://osf.io/gesh7/).

## References

[1] Jimenez, A. G. The physiological conundrum that is the domestic dog. *Integr. Comp. Biol.* **61**, 140–153 (2021). 33705526 10.1093/icb/icab005

[2] Nagasawa, M. et al. Oxytocin-gaze positive loop and the Coevolution of human-dog bonds. *Science* **348**, 333–336 (2015). 25883356 10.1126/science.1261022

[3] Scott, J. P. The effects of selection and domestication upon the behavior of the Dog12. *JNCI J. Natl. Cancer Inst.* **15**, 739–758 (1954). 13233923

[4] vonHoldt, B. M. et al. Structural variants in genes associated with human Williams-Beuren syndrome underlie stereotypical hypersociability in domestic dogs. *Sci. Adv.* **3**, e1700398 (2017). 28776031 10.1126/sciadv.1700398 PMC5517105

[5] Bray, E. E. et al. Early-emerging and highly heritable sensitivity to human communication in dogs. *Curr. Biol.* **31**, 3132–3136e5 (2021). 34087106 10.1016/j.cub.2021.04.055

[6] Schneider, A. K. E. & Bräuer, J. Exploring levels of interspecies interaction: expectations, knowledge, and empathy in Human–Dog relationships. *Animals* **14**, 2509 (2024). 39272293 10.3390/ani14172509 PMC11394575

[7] Paul, M., Sen Majumder, S., Sau, S., Nandi, A. K. & Bhadra, A. High early life mortality in free-ranging dogs is largely influenced by humans. *Sci. Rep.* **6**, 19641 (2016). 26804633 10.1038/srep19641 PMC4726281

[8] Kwan, J. Y. & Bain, M. J. Owner attachment and problem behaviors related to relinquishment and training techniques of dogs. *J. Appl. Anim. Welf. Sci.* **16**, 168–183 (2013). 23544756 10.1080/10888705.2013.768923

[9] Powell, L., Duffy, D. L., Kruger, K. A., Watson, B. & Serpell, J. A. Relinquishing owners underestimate their dog’s behavioral problems: deception or lack of knowledge?? *Front Vet. Sci* **8**, 734973 (2021). 10.3389/fvets.2021.734973 PMC8461173 34568478

[10] Hitchcock, M., Workman, M. K., Guthrie, A. P. & Ruple, A. & Feuerbacher, E. N. Factors associated with behavioral euthanasia in pet dogs. *Front Vet. Sci* **11**, 1387076 (2024). 10.3389/fvets.2024.1387076 PMC11091869 38746931

[11] Boardman, H. & Farnworth, M. J. Changes to adult dog social behaviour during and after COVID-19 lockdowns in england: A qualitative analysis of owner perception. *Animals* **12**, 1682 (2022). 35804581 10.3390/ani12131682 PMC9264766

[12] Habarth-Morales, T. E., Rios-Diaz, A. J. & Caterson, E. J. Pandemic puppies: man’s best friend or public health problem?? A multidatabase study. *J. Surg. Res.* **276**, 203–207 (2022). 35378364 10.1016/j.jss.2022.02.041 PMC9576631

[13] Plana, N. M., Kalmar, C. L., Cheung, L., Swanson, J. W. & Taylor, J. A. Pediatric dog bite injuries: A 5-Year nationwide study and implications of the COVID-19 pandemic. *J. Craniofac. Surg.* **33**, 1436 (2022). 35758430 10.1097/SCS.0000000000008670 PMC9275798

[14] Sacchettino, L. et al. Puppies Raised during the COVID-19 lockdown showed fearful and aggressive behaviors in adulthood: an Italian survey. *Vet. Sci.* **10**, 198 (2023). 36977237 10.3390/vetsci10030198 PMC10059587

[15] Sherwell, E. G. et al. Changes in dog behaviour associated with the COVID-19 lockdown, Pre-Existing Separation-Related problems and alterations in owner behaviour. *Vet. Sci.* **10**, 195 (2023). 36977234 10.3390/vetsci10030195 PMC10059576

[16] Nemeroff, C. B. Paradise lost: the Neurobiological and clinical consequences of child abuse and neglect. *Neuron* **89**, 892–909 (2016). 26938439 10.1016/j.neuron.2016.01.019

[17] Green, J. G. et al. Childhood adversities and adult psychiatric disorders in the National comorbidity survey replication I: associations with first onset of DSM-IV disorders. *Arch. Gen. Psychiatry*. **67**, 113–123 (2010). 20124111 10.1001/archgenpsychiatry.2009.186 PMC2822662

[18] Norman, R. E. et al. The Long-Term health consequences of child physical abuse, emotional abuse, and neglect: A systematic review and Meta-Analysis. *PLOS Med.* **9**, e1001349 (2012). 23209385 10.1371/journal.pmed.1001349 PMC3507962

[19] Gunnar, M. R. Early adversity, stress, and neurobehavioral development. *Dev. Psychopathol.* **32**, 1555–1562 (2020). 33427166 10.1017/S0954579420001649

[20] Ainsworth, M. D. S., Blehar, M. C., Waters, E. & Wall, S. N. *Patterns of Attachment: A Psychological Study of the Strange Situation* (Psychology, 2015). 10.4324/9780203758045

[21] Sánchez, M. M., Ladd, C. O. & Plotsky, P. M. Early adverse experience as a developmental risk factor for later psychopathology: evidence from rodent and primate models. *Dev. Psychopathol.* **13**, 419–449 (2001). 11523842 10.1017/s0954579401003029

[22] Pryce, C. R., Dettling, A. C., Spengler, M., Schnell, C. R. & Feldon, J. Deprivation of parenting disrupts development of homeostatic and reward systems in marmoset monkey offspring. *Biol. Psychiatry*. **56**, 72–79 (2004). 15231438 10.1016/j.biopsych.2004.05.002

[23] Spinelli, S. et al. Early-Life stress induces Long-term morphologic changes in primate brain. *Arch. Gen. Psychiatry*. **66**, 658–665 (2009). 19487631 10.1001/archgenpsychiatry.2009.52 PMC3873603

[24] Stevens, H. E., Leckman, J. F., Coplan, J. D. & Suomi, S. J. Risk and resilience: early manipulation of macaque social experience and persistent behavioral and neurophysiological outcomes. *J. Am. Acad. Child. Adolesc. Psychiatry*. **48**, 114–127 (2009). 19127170 10.1097/CHI.0b013e318193064c

[25] McPhedran, S. & Animal Abuse Family violence, and child wellbeing: A review. *J. Fam Violence*. **24**, 41–52 (2009).

[26] McDonald, S. E. et al. Children’s experiences of companion animal maltreatment in households characterized by intimate partner violence. *Child. Abuse Negl.* **50**, 116–127 (2015). 26520828 10.1016/j.chiabu.2015.10.005 PMC4688106

[27] Campbell, A. M. The intertwined Well-Being of children and Non-Human animals: an analysis of animal control reports involving children. *Soc. Sci.* **11**, 46 (2022).

[28] Buttner, A. P. & Strasser, R. Extreme life histories are associated with altered social behavior and cortisol levels in shelter dogs. *Appl. Anim. Behav. Sci.* **256**, 105693 (2022).

[29] McMillan, F. D., Duffy, D. L., Zawistowski, S. L. & Serpell, J. A. Behavioral and psychological characteristics of canine victims of abuse. *J. Appl. Anim. Welf. Sci.* **18**, 92–111 (2015). 25257564 10.1080/10888705.2014.962230

[30] McMillan, F. D., Vanderstichel, R., Stryhn, H., Yu, J. & Serpell, J. A. Behavioural characteristics of dogs removed from hoarding situations. *Appl. Anim. Behav. Sci.* **178**, 69–79 (2016).

[31] Miller, K. A., Dolan, E. D., Cussen, V. A. & Reid, P. J. Are Underweight Shelter Dogs More Likely to Display Food Aggression toward Humans? *Animals* 9, 1035 (2019). 10.3390/ani9121035 PMC6940832 31783499

[32] Foyer, P., Bjällerhag, N., Wilsson, E. & Jensen, P. Behaviour and experiences of dogs during the first year of life predict the outcome in a later temperament test. *Appl. Anim. Behav. Sci.* **155**, 93–100 (2014).

[33] Serpell, J. A. & Duffy, D. L. Aspects of juvenile and adolescent environment predict aggression and fear in 12-Month-Old guide dogs. *Front. Vet. Sci.* **3**, 49 (2016). 27446937 10.3389/fvets.2016.00049 PMC4916180

[34] Bray, E. E., Sammel, M. D., Cheney, D. L., Serpell, J. A. & Seyfarth, R. M. Effects of maternal investment, temperament, and cognition on guide dog success. *Proc. Natl. Acad. Sci.* 114, 9128–9133 (2017). 10.1073/pnas.1704303114 PMC5576795 28784785

[35] Fox, M. W. & Stelzner, D. The effects of early experience on the development of inter and intraspecies social relationships in the dog. *Anim. Behav.* **15**, 377–386 (1967). 6030963 10.1016/0003-3472(67)90024-3

[36] Appleby, D. L., Bradshaw, J. W. S. & Casey, R. A. Relationship between aggressive and avoidance behaviour by dogs and their experience in the first six months of life. *Vet. Rec*. **150**, 434–438 (2002). 11993972 10.1136/vr.150.14.434

[37] Stoltenborgh, M., Bakermans-Kranenburg, M. J., Alink, L. R. A. & van IJzendoorn, M. H. The prevalence of child maltreatment across the globe: review of a series of Meta-Analyses. *Child. Abuse Rev.* **24**, 37–50 (2015).

[38] Kessler, R. C., Ruscio, A. M., Shear, K. & Wittchen, H. U. Epidemiology of anxiety disorders. In *Behavioral Neurobiology of Anxiety and its Treatment* (eds Stein, M. B. & Steckler, T.) 21–35 (Springer, 2010). 10.1007/7854_2009_9. 21309104

[39] Remes, O., Brayne, C., van der Linde, R. & Lafortune, L. A systematic review of reviews on the prevalence of anxiety disorders in adult populations. *Brain Behav.* **6**, e00497 (2016). 27458547 10.1002/brb3.497 PMC4951626

[40] FCI Breeds Nomenclature. https://fci.be/en/Nomenclature/

[41] Callaghan, B. L. & Tottenham, N. The stress acceleration hypothesis: effects of early-life adversity on emotion circuits and behavior. *Curr. Opin. Behav. Sci.* **7**, 76–81 (2016). 29644262 10.1016/j.cobeha.2015.11.018 PMC5890821

[42] Heim, C. & Binder, E. B. Current research trends in early life stress and depression: review of human studies on sensitive periods, gene–environment interactions, and epigenetics. *Exp. Neurol.* **233**, 102–111 (2012). 22101006 10.1016/j.expneurol.2011.10.032

[43] Nusslock, R. & Miller, G. E. Early-Life adversity and physical and emotional health across the lifespan: A neuroimmune network hypothesis. *Biol. Psychiatry*. **80**, 23–32 (2016). 26166230 10.1016/j.biopsych.2015.05.017 PMC4670279

[44] Smith, K. E. & Pollak, S. D. Early life stress and development: potential mechanisms for adverse outcomes. *J. Neurodev Disord*. **12**, 34 (2020). 33327939 10.1186/s11689-020-09337-y PMC7745388

[45] Howell, B. R. et al. Early adverse experience increases emotional reactivity in juvenile rhesus macaques: relation to amygdala volume. *Dev. Psychobiol.* **56**, 1735–1746 (2014). 25196846 10.1002/dev.21237 PMC4433484

[46] O’Mahony, S. M. et al. Early life stress alters behavior, immunity, and microbiota in rats: implications for irritable bowel syndrome and psychiatric illnesses. *Biol. Psychiatry*. **65**, 263–267 (2009). 18723164 10.1016/j.biopsych.2008.06.026

[47] Uchida, S. et al. Early life stress enhances behavioral vulnerability to stress through the activation of REST4-Mediated gene transcription in the medial prefrontal cortex of rodents. *J. Neurosci.* **30**, 15007–15018 (2010). 21068306 10.1523/JNEUROSCI.1436-10.2010 PMC6633839

[48] Buttner, A. P., Awalt, S. L. & Strasser, R. Early life adversity in dogs produces altered physiological and behavioral responses during a social stress-buffering paradigm. *J. Exp. Anal. Behav.* **120**, 6–20 (2023). 37210677 10.1002/jeab.856

[49] Foyer, P., Wilsson, E., Wright, D. & Jensen, P. Early experiences modulate stress coping in a population of German shepherd dogs. *Appl. Anim. Behav. Sci.* **146**, 79–87 (2013).

[50] Duffy, D. L., Hsu, Y. & Serpell, J. A. Breed differences in canine aggression. *Appl. Anim. Behav. Sci.* **114**, 441–460 (2008).

[51] Zapata, I., Serpell, J. A. & Alvarez, C. E. Genetic mapping of canine fear and aggression. *BMC Genom.* **17**, 572 (2016). 10.1186/s12864-016-2936-3 PMC4977763 27503363

[52] Zapata, I., Lilly, M. L., Herron, M. E., Serpell, J. A. & Alvarez, C. E. Genetic testing of dogs predicts problem behaviors in clinical and nonclinical samples. *BMC Genom.* **23**, 102 (2022). 10.1186/s12864-022-08351-9 PMC8819838 35130840

[53] Mehrkam, L. R. & Wynne, C. D. L. Behavioral differences among breeds of domestic dogs (Canis lupus familiaris): current status of the science. *Appl. Anim. Behav. Sci.* **155**, 12–27 (2014).

[54] Wormald, D., Lawrence, A. J., Carter, G. & Fisher, A. D. Analysis of correlations between early social exposure and reported aggression in the dog. *J. Vet. Behav.* **15**, 31–36 (2016).

[55] Dietz, L., Arnold, A. M. K., Goerlich-Jansson, V. C. & Vinke, C. M. The importance of early life experiences for the development of behavioural disorders in domestic dogs. (2018). 10.1163/1568539X-00003486

[56] Gillespie, C. F., Phifer, J., Bradley, B. & Ressler, K. J. Risk and resilience: genetic and environmental influences on development of the stress response. *Depress. Anxiety*. **26**, 984–992 (2009). 19750552 10.1002/da.20605 PMC2852579

[57] Casey, B. et al. The storm and stress of adolescence: insights from human imaging and mouse genetics. *Dev. Psychobiol.* **52**, 225–235 (2010). 20222060 10.1002/dev.20447 PMC2850961

[58] Dion, A., Muñoz, P. T. & Franklin, T. B. Epigenetic mechanisms impacted by chronic stress across the rodent lifespan. *Neurobiol. Stress*. **17**, 100434 (2022). 35198660 10.1016/j.ynstr.2022.100434 PMC8841894

[59] Peña, C. J. et al. Early life stress alters transcriptomic patterning across reward circuitry in male and female mice. *Nat. Commun.* **10**, 5098 (2019). 31704941 10.1038/s41467-019-13085-6 PMC6841985

[60] Fairbanks, L. A. et al. Heritability and genetic correlation of hair cortisol in Vervet monkeys in low and higher stress environments. *Psychoneuroendocrinology* **36**, 1201–1208 (2011). 21411232 10.1016/j.psyneuen.2011.02.013 PMC3125414

[61] Holmes, A. et al. Early life genetic, epigenetic and environmental factors shaping emotionality in rodents. *Neurosci. Biobehav Rev.* **29**, 1335–1346 (2005). 16095695 10.1016/j.neubiorev.2005.04.012

[62] Santarelli, S. et al. An adverse early life environment can enhance stress resilience in adulthood. *Psychoneuroendocrinology* **78**, 213–221 (2017). 28219813 10.1016/j.psyneuen.2017.01.021

[63] Bonanno, G. A., Westphal, M. & Mancini, A. D. Resilience to loss and potential trauma. *Annu. Rev. Clin. Psychol.* **7**, 511–535 (2011). 21091190 10.1146/annurev-clinpsy-032210-104526

[64] Radley, J. J. & Herman, J. P. Preclinical models of chronic stress: adaptation or pathology?? *Biol. Psychiatry*. **94**, 194–202 (2023). 36631383 10.1016/j.biopsych.2022.11.004 PMC10166771

[65] Scott, J. P. & Marston, M. V. Critical periods affecting the development of normal and Mal-Adjustive social behavior of puppies. *Pedagog Semin J. Genet. Psychol*. **77**(1), 25–60 (1950). 10.1080/08856559.1950.10533536 14794866

[66] Scott, J. P. & Fuller, J. L. *Genetics and the Social Behavior of the Dog* (University of Chicago Press, 2012).

[67] Fox, M. W. Socio-Ecological implications of individual differences in Wolf litters: a developmental and evolutionary perspective. (1972). 10.1163/156853972X00077

[68] Scott, J. P. The evolution of social behavior in dogs and wolves. *Am. Zool.* **7**, 373–381 (1967).

[69] Bray, E. E. et al. Cognitive characteristics of 8- to 10-week-old assistance dog puppies. *Anim. Behav.* **166**, 193–206 (2020). 32719570 10.1016/j.anbehav.2020.05.019 PMC7384752

[70] Foyer, P., Wilsson, E. & Jensen, P. Levels of maternal care in dogs affect adult offspring temperament. *Sci. Rep.* **6**, 19253 (2016). 26758076 10.1038/srep19253 PMC4725833

[71] Bray, E. E. et al. Enhancing the selection and performance of working dogs. *Front Vet. Sci* **8**, 644431 (2021). 10.3389/fvets.2021.644431 PMC8149746 34055947

[72] McGreevy, P. D. et al. Dog behavior Co-Varies with height, bodyweight and skull shape. *PLOS ONE*. **8**, e80529 (2013). 24358107 10.1371/journal.pone.0080529 PMC3864788

[73] MacLean, E. L., Snyder-Mackler, N., vonHoldt, B. M. & Serpell, J. A. Highly heritable and functionally relevant breed differences in dog behaviour. *Proc. R. Soc. B Biol. Sci.* 286, 20190716 (2019). 10.1098/rspb.2019.0716 PMC6790757 31575369

[74] Reisner, I. R., Shofer, F. S. & Nance, M. L. Behavioral assessment of child-directed canine aggression. *Inj Prev.* **13**, 348–351 (2007). 17916894 10.1136/ip.2007.015396 PMC2610618

[75] Casey, R. A., Loftus, B., Bolster, C., Richards, G. J. & Blackwell, E. J. Human directed aggression in domestic dogs (*Canis familiaris*): occurrence in different contexts and risk factors. *Appl. Anim. Behav. Sci.* **152**, 52–63 (2014).

[76] Nagasawa, M., Murai, K., Mogi, K. & Kikusui, T. Dogs can discriminate human smiling faces from blank expressions. *Anim. Cogn.* **14**, 525–533 (2011). 21359654 10.1007/s10071-011-0386-5

[77] Tamimi, N., Jamshidi, S., Serpell, J. A., Mousavi, S. & Ghasempourabadi, Z. Assessment of the C-BARQ for evaluating dog behavior in Iran. *J. Vet. Behav.* **10**, 36–40 (2015).

[78] Hsu, Y. & Serpell, J. A. Development and validation of a questionnaire for measuring behavior and temperament traits in pet dogs. (2003). 10.2460/javma.2003.223.1293 10.2460/javma.2003.223.1293 14621216

[79] Ghosh-Ippen, C. et al. Traumatic Events Screening Inventory-Parent Report Revised-Long Version. (2014). 10.1037/t30813-000

[80] Duffy, D. L. & Serpell, J. A. Predictive validity of a method for evaluating temperament in young guide and service dogs. *Appl. Anim. Behav. Sci.* **138**, 99–109 (2012).

[81] Parker, H. G. et al. Genomic analyses reveal the influence of geographic origin, migration, and hybridization on modern dog breed development. *Cell. Rep.* **19**, 697–708 (2017). 28445722 10.1016/j.celrep.2017.03.079 PMC5492993

[82] R Core Team. *R: A Language and Environment for Statistical Computing*. (2023). https://www.R-project.org/

