# Supplementary material for: Influence of early life adversity and breed on aggression and fear in dogs
Source: Sci Rep. 2025 Oct 2;15:32590. doi: 10.1038/s41598-025-18226-0 (PMC12491534; doi:10.1038/s41598-025-18226-0)
Supplement: Supplementary file 1 — Supplementary Material 1 [file 41598_2025_18226_MOESM1_ESM.docx]

**Supporting Information**

**Supporting Analyses**

***Main result with FCI breed classification***

Given the insufficient breed relatedness data available to include all breeds in this work, we categorized breeds into groups according to the Fédération Cynologique Internationale (*FCI Breeds Nomenclature*) classification system. This approach allowed for a standardized and widely accepted framework for breed categorization and facilitated analysis and interpretation of the results. Breed groups include: i- Sheepdogs and cattle dogs; ii - Pinschers and schnauzers; iii - Terriers; iv - Dachshunds; v - Spitz and primitive types; vi - Scent hounds; vii - Pointing dogs; viii - Retrievers; ix - Companion and toy dogs; x - Sight hounds. We categorized breeds that are not recognized under the FCI as “Non-FCI breeds,” and dogs of multiple breed or uncertain genetic ancestry as “Mixed breed” (see Table S2-S3 for detailed demographic information of the sample). Results of the main model using genetic clade groups (Table 1) are identical to the results of the model variation using FCI breed groups (Table S1).

Table S1. Contribution of heritable and environmental factors on perceived aggression and fear, with FCI breed group classification.

|  | | Aggression | | | Fear | | |
| --- | --- | --- | --- | --- | --- | --- | --- |
| Covariate | *F* | | *p-value* | *r* | *F* | *p-value* | *r* |
| Breed group | 9.9 | | <.001 | .155 | 7.03 | <.001 | .131 |
| Weight (kg) | 55.0 | | <.001 | .111 | 103.1 | <.001 | .150 |
| Sex | 35.1 | | <.001 | .088 | 0.4 | .512 | .010 |
| Age (years) | 19.0 | | <.001 | .076 | 0.3 | .579 | .022 |
| Desexed (yes/no) | 0.3 | | .608 | .008 | 5.3 | .022 | .034 |
| Cohabit with child (yes/no) | 130.8 | | <.001 | .171 | 31.9 | <.001 | .088 |
| Source of acquisition (breeder/not-breeder) | 92.3 | | <.001 | .142 | 74.2 | <.001 | .129 |
| Early adversity (yes/no) | 28.0 | | <.001 | .079 | 41.4 | <.001 | .096 |
| Cohabit with dog (yes/no) | 9.0 | | .003 | .053 | 0.0 | .989 | .017 |
| Exercise (hrs) | 2.7 | | .101 | .022 | 6.5 | .011 | .038 |

*Note*. Estimates of effects obtained from *car* package *(*Version 3.1-2; *Fox & Weisberg, 2019).* Numerator *df* is 11 for breed group and 1 for all other covariates. *F =* test statistic of Type II Anova. *r* = effect size obtained by taking the square root from the partial *R*^2^ estimates of each covariate from the *jtools* package (Version 2.2.2; *Long, 2022*).

***Subscales of aggression and fear***

Beyond the pre-registered hypotheses, we explored the impact of early life adversity on specific types of aggressive and fear behaviour. The C-BARQ includes five factors for dog aggression (unfamiliar human aggression, familiar human aggression, unfamiliar dog aggression, familiar dog aggression, predatory aggression) and three factors of fear (unfamiliar human fear, unfamiliar dog fear, nonsocial fear). In exploratory analyses, we evaluated the fit of the theoretically postulated measurement model with confirmatory factor analysis in *lavaan* (Rosseel, 2012). Because the model fit was acceptable, CFI = 0.863, TLI = 0.846, RMSEA = 0.053, we used this model for subsequent structural equation modeling analyses where we regressed respective factors on early life adversity, while controlling for the same set of covariates as in our main analyses, and allowing for intercorrelations between aggression and fear factors. The latter step allowed us to control for some forms of response bias, simultaneously evaluating unique effects of early life adversity on the respective factor when accounting for shared variance with the other factors in the same model. The results indicated a significant contribution of early life adversity for unfamiliar human aggression, β = 0.090, *z* = 6.30, *p* < .001, familiar human aggression, β = 0.049, *z* = 3.51, *p* < .001, familiar dog aggression, β = 0.054, *z* = 3.34, *p* = .001, unfamiliar human fear, β = 0.117, *z* = 7.77, *p* < .001, unfamiliar dog fear, β = 0.041, *z* = 2.67, *p* = .008, and non-social fear, β = 0.090, *z* = 5.63, *p* < .001, but not for unfamiliar dog aggression, β = 0.018, *z* = 1.28, *p* = .202, suggesting that human-directed aggression and fear is influenced by a broad range of adverse experiences (abuse, lacking a primary caregiver) while dog-directed aggression may be more selectively influenced.

***Impact of adversity by breed on aggression and fear***

Exploratory analyses of the impact of adverse early life history by breed on behaviour with a minimum of 10 dogs per condition within each breed revealed nearly identical results as were found with the lower threshold of min *n* = 5 dogs per condition. Breeds that showed a significantly greater distance in mean scores after adversity included Airedale Terriers (fear: *t*(1348) = 2.40, *p* =.017), Golden Retrievers (fear: *t*(1348) = 2.03, *p* =.043), and American Eskimo Dogs (aggression: *t*(1348) = 2.30, *p* =.021; fear *t*(1348) = 2.13, *p* =.033).

*Figure S1. Average aggression by breed and history of adversity,* min *n /* cell = 10.


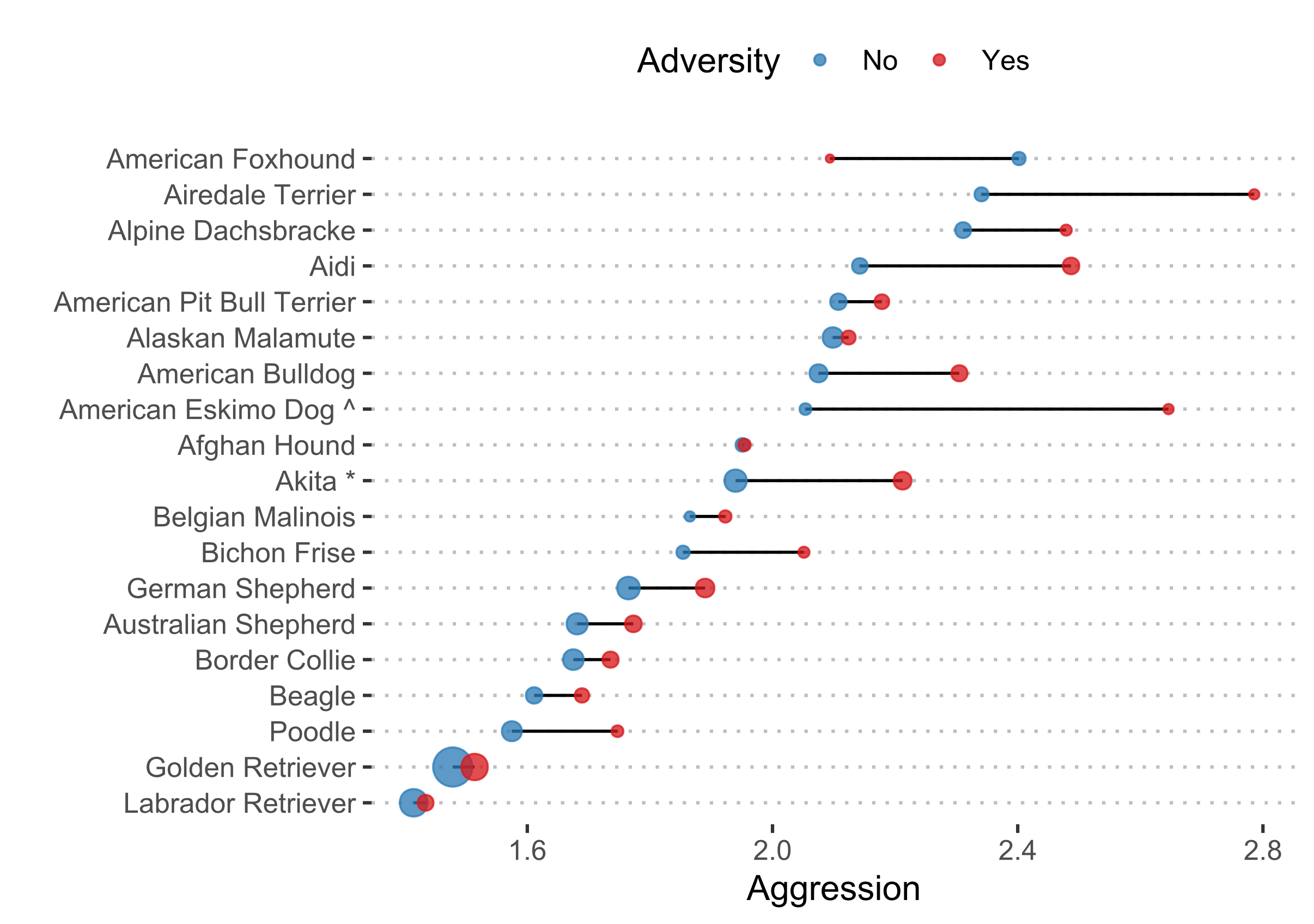


*Figure S2. Average fear by breed and history of adversity,* min *n /* cell = 10.

*
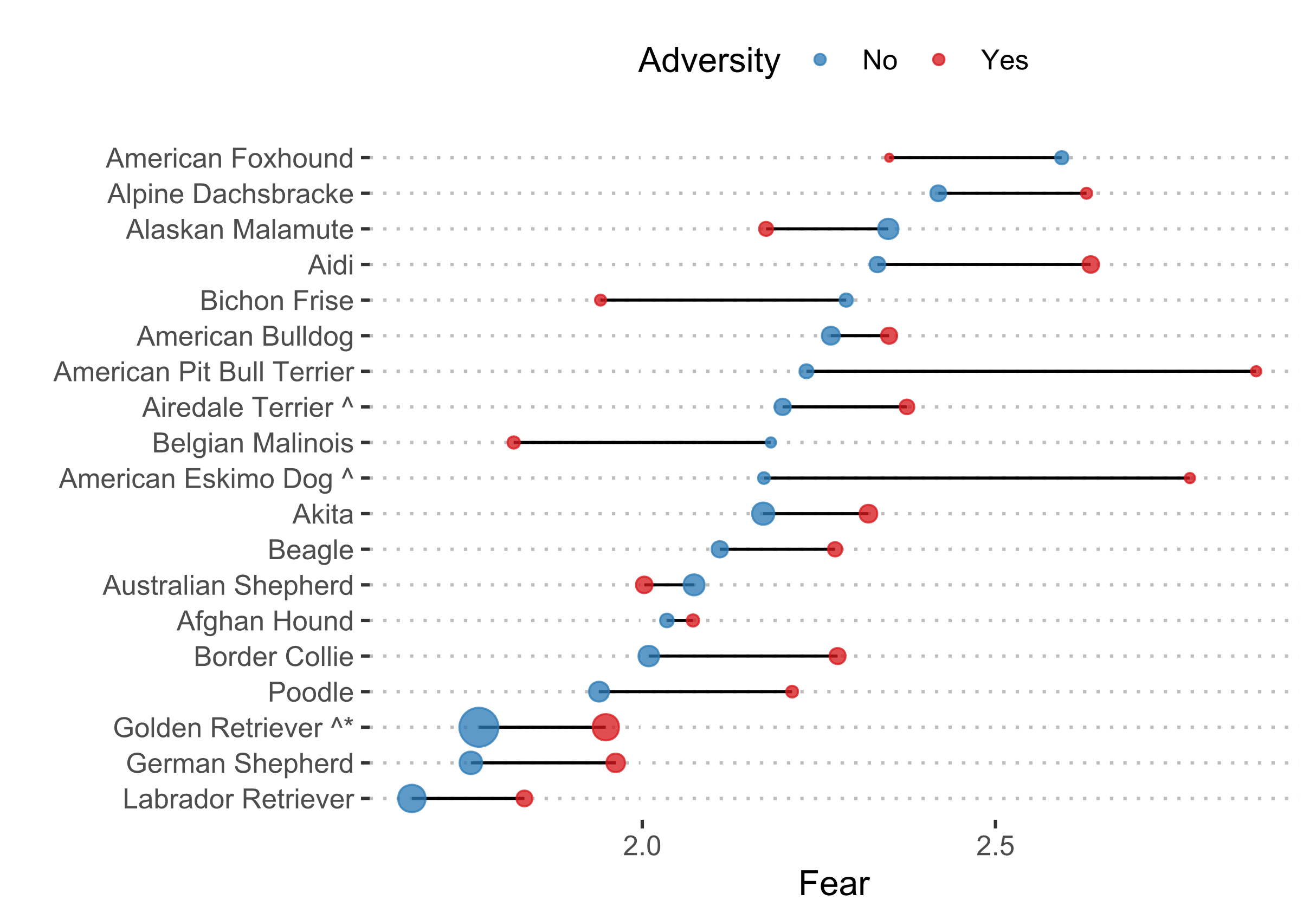
*

**Sample Demographics**

Table S2. Breed distribution for all dogs in the sample with genetic clade (Parker et al., 2017) and FCI classification (*FCI Breeds Nomenclature*).

| **Breed** | | **Clade** | | **FCI Class** | **Female : Male** | **N_**  **Adversity** |
| --- | --- | --- | --- | --- | --- | --- |
| Affenpinscher | | NonClade | | PinscherSchnauzer | 0 : 14 | 7 |
| Afghan Hound | | Mediterranean | | Sighthound | 7 : 26 | 15 |
| Aidi | | NonClade | | PinscherSchnauzer | 17 : 35 | 28 |
| Airedale Terrier | | Terrier | | Terrier | 6 : 25 | 12 |
| Akbash | | NonClade | | NonFCIBreed | 6 : 23 | 9 |
| Akita (American/Japanese) | | Asian Spitz | | SpitzPrimitive | 25 : 67 | 33 |
| Alapaha Blue-Blood Bulldog | | NonClade | | NonFCIBreed | 0 : 1 | 0 |
| Alaskan Klee Kai | | NonClade | | SpitzPrimitive | 9 : 30 | 9 |
| Alaskan Malamute | | Asian Spitz | | SpitzPrimitive | 22 : 44 | 19 |
| Alpine Dachsbracke | | NonClade | | Scenthound | 12 : 26 | 13 |
| American Bulldog | | NonClade | | NonFCIBreed | 18 : 42 | 26 |
| American Bully | | NonClade | | NonFCIBreed | 1 : 2 | 2 |
| American English Coonhound | | NonClade | | NonFCIBreed | 5 : 21 | 5 |
| American Eskimo Dog (Standard/Toy/Miniature) | | Nordic Spitz | | NonFCIBreed | 8 : 18 | 12 |
| American Foxhound | | NonClade | | NonFCIBreed | 12 : 16 | 11 |
| American Hairless Terrier | | American Terrier | | NonFCIBreed | 6 : 13 | 10 |
| American Leopard Hound | | NonClade | | NonFCIBreed | 6 : 8 | 5 |
| American Pit Bull Terrier | | NonClade | | NonFCIBreed | 10 : 38 | 21 |
| American Staffordshire Terrier | | European Mastiff | | Terrier | 1 : 0 | 0 |
| American Water Spaniel | | NonClade | | RetrieverFlushingWater | 0 : 4 | 1 |
| Anatolian Shepherd Dog | | Mediterranean | | PinscherSchnauzer | 3 : 11 | 5 |
| Appenzeller Sennenhunde | | NonClade | | PinscherSchnauzer | 2 : 2 | 2 |
| Ariegeois | | NonClade | | Scenthound | 1 : 0 | 0 |
| Armenian Gampr | | NonClade | | NonFCIBreed | 0 : 1 | 0 |
| Australian Cattle Dog (Standard/Stumpy Tail) | | UK Rural | | SheepdogCattledog | 9 : 13 | 7 |
| Australian Kelpie | | NonClade | | SheepdogCattledog | 2 : 0 | 0 |
| Australian Shepherd (Standard/Miniature) | | UK Rural | | SheepdogCattledog | 29 : 51 | 28 |
| Austrian Black and Tan Hound | | NonClade | | Scenthound | 1 : 1 | 2 |
| Austrian Pinscher | | NonClade | | PinscherSchnauzer | 0 : 2 | 1 |
| Azawakh | | Mediterranean | | Sighthound | 0 : 1 | 1 |
| Barak (Bosnian Coarse-haired Hound) | | NonClade | | Scenthound | 1 : 2 | 0 |
| Barbet | | NonClade | | RetrieverFlushingWater | 3 : 3 | 1 |
| Basenji | | NonClade | | SpitzPrimitive | 1 : 0 | 0 |
| Basset Hound | | Scent Hound | | Scenthound | 2 : 1 | 2 |
| Bavarian Mountain Hound | | NonClade | | Scenthound | 1 : 2 | 1 |
| Beagle | | Scent Hound | | Scenthound | 18 : 29 | 20 |
| Beagle Harrier | | NonClade | | Scenthound | 2 : 3 | 2 |
| Bearded Collie | | UK Rural | | SheepdogCattledog | 5 : 2 | 2 |
| Beauceron | | NonClade | | SheepdogCattledog | 1 : 0 | 0 |
| Bedlington Terrier | | Terrier | | Terrier | 4 : 3 | 3 |
| Belgian Laekenois | | NonClade | | SheepdogCattledog | 0 : 2 | 0 |
| Belgian Malinois | | Continental Herder | | SheepdogCattledog | 13 : 14 | 15 |
| Belgian Sheepdog | | Continental Herder | | SheepdogCattledog | 3 : 5 | 2 |
| Belgian Tervuren | | Continental Herder | | SheepdogCattledog | 1 : 2 | 1 |
| Berger Blanc Suisse | | NonClade | | SheepdogCattledog | 2 : 0 | 0 |
| Berger Picard | | New World | | SheepdogCattledog | 0 : 1 | 0 |
| Bernese Mountain Dog | | Alpine | | PinscherSchnauzer | 6 : 7 | 4 |
| Bichon Frise | | Poodle | | CompanionToy | 8 : 22 | 13 |
| Billy | | NonClade | | Scenthound | 0 : 3 | 0 |
| Black Mouth Cur | | NonClade | | NonFCIBreed | 1 : 1 | 1 |
| Black Russian Terrier | | Drover | | PinscherSchnauzer | 2 : 1 | 2 |
| Bloodhound | | Scent Hound | | Scenthound | 3 : 1 | 1 |
| Bluetick Coonhound | | NonClade | | NonFCIBreed | 0 : 2 | 0 |
| Boerboel | | European Mastiff | | NonFCIBreed | 1 : 0 | 0 |
| Border Collie | | UK Rural | | SheepdogCattledog | 34 : 40 | 26 |
| Border Terrier | | Terrier | | Terrier | 5 : 9 | 1 |
| Borzoi | | UK Rural | | Sighthound | 0 : 3 | 1 |
| Boston Terrier | | European Mastiff | | CompanionToy | 5 : 6 | 2 |
| Bouvier des Ardennes | | NonClade | | SheepdogCattledog | 0 : 1 | 0 |
| Bouvier des Flandres | | Continental Herder | | SheepdogCattledog | 7 : 5 | 2 |
| Boxer | | European Mastiff | | PinscherSchnauzer | 4 : 8 | 2 |
| Bracco Italiano | | NonClade | | Pointing | 1 : 1 | 1 |
| Braque de l'Ariege (Ariege Pointer) | | NonClade | | Pointing | 0 : 2 | 0 |
| Braque du Bourbonnais (Bourbonnais Pointer) | | NonClade | | Pointing | 0 : 1 | 0 |
| Briard | | Continental Herder | | SheepdogCattledog | 0 : 1 | 0 |
| Brittany (American/French) | | Pointer Setter | | Pointing | 2 : 3 | 0 |
| Broholmer (Danish Broholmer) | | NonClade | | PinscherSchnauzer | 1 : 0 | 0 |
| Brussels Griffon | | Toy Spitz | | CompanionToy | 1 : 3 | 0 |
| Bull Terrier (Standard/Miniature) | | European Mastiff | | Terrier | 2 : 1 | 0 |
| Bulldog | | European Mastiff | | PinscherSchnauzer | 2 : 5 | 0 |
| Bullmastiff | | European Mastiff | | PinscherSchnauzer | 1 : 1 | 1 |
| Cairn Terrier | | Terrier | | Terrier | 1 : 0 | 0 |
| Canaan Dog | | NonClade | | SpitzPrimitive | 1 : 0 | 0 |
| Canadian Eskimo Dog | | NonClade | | SpitzPrimitive | 0 : 2 | 0 |
| Cane Corso | | European Mastiff | | PinscherSchnauzer | 2 : 5 | 2 |
| Cao de Castro Laboreiro | | NonClade | | PinscherSchnauzer | 1 : 0 | 0 |
| Cardigan Welsh Corgi | | UK Rural | | SheepdogCattledog | 0 : 4 | 1 |
| Caucasian Ovcharka | | NonClade | | PinscherSchnauzer | 0 : 1 | 1 |
| Cavalier King Charles Spaniel | | Spaniel | | CompanionToy | 4 : 8 | 0 |
| Central Asian Shepherd Dog | | NonClade | | PinscherSchnauzer | 2 : 1 | 1 |
| Chart Polski (Polish Greyhound) | | NonClade | | Sighthound | 0 : 1 | 0 |
| Chesapeake Bay Retriever | | NonClade | | RetrieverFlushingWater | 1 : 0 | 1 |
| Chihuahua | | American Toy | | CompanionToy | 7 : 13 | 3 |
| Chinese Crested Dog | | American Toy | | CompanionToy | 3 : 2 | 1 |
| Chinese Shar Pei (Standard/Miniature) | | Asian Spitz | | PinscherSchnauzer | 1 : 2 | 0 |
| Chow Chow | | Asian Spitz | | SpitzPrimitive | 0 : 1 | 0 |
| Clumber Spaniel | | NonClade | | RetrieverFlushingWater | 0 : 1 | 0 |
| Cocker Spaniel (American) | | NonClade | | RetrieverFlushingWater | 3 : 5 | 2 |
| Collie (Rough/Smooth) | | UK Rural | | SheepdogCattledog | 5 : 5 | 2 |
| Coton de Tulear | | Poodle | | CompanionToy | 2 : 3 | 1 |
| Dachshund (Longhaired/Smooth/Wirehaired) | | Scent Hound | | Dachshund | 9 : 21 | 9 |
| Dalmatian | | Pointer Setter | | Scenthound | 5 : 3 | 3 |
| Deutsche Bracke | | NonClade | | Scenthound | 0 : 1 | 1 |
| Doberman Pinscher | | Drover | | PinscherSchnauzer | 8 : 4 | 2 |
| Drever | | NonClade | | Scenthound | 0 : 2 | 1 |
| Dutch Shepherd | | NonClade | | SheepdogCattledog | 1 : 2 | 0 |
| English Cocker Spaniel | | Spaniel | | RetrieverFlushingWater | 8 : 3 | 1 |
| English Setter | | Pointer Setter | | Pointing | 4 : 0 | 2 |
| English Shepherd | | NonClade | | NonFCIBreed | 3 : 7 | 5 |
| English Springer Spaniel | | Spaniel | | RetrieverFlushingWater | 11 : 9 | 3 |
| English Toy Spaniel | | NonClade | | CompanionToy | 1 : 1 | 1 |
| English Toy Terrier (Black and Tan) | | NonClade | | Terrier | 0 : 1 | 1 |
| Entlebucher Mountain Dog | | NonClade | | PinscherSchnauzer | 1 : 1 | 1 |
| Eurasier | | NonClade | | SpitzPrimitive | 1 : 0 | 0 |
| Field Spaniel | | Spaniel | | RetrieverFlushingWater | 0 : 1 | 1 |
| Flat-Coated Retriever | | Retriever | | RetrieverFlushingWater | 1 : 1 | 0 |
| French Bulldog | | European Mastiff | | CompanionToy | 4 : 3 | 2 |
| German Longhaired Pointer | | NonClade | | Pointing | 1 : 0 | 0 |
| German Shepherd Dog | | New World | | SheepdogCattledog | 44 : 54 | 37 |
| German Shorthaired Pointer | | Pointer Setter | | Pointing | 3 : 5 | 3 |
| German Wirehaired Pointer | | Pointer Setter | | Pointing | 1 : 0 | 1 |
| Glen Imaal Terrier | | Terrier | | Terrier | 2 : 2 | 0 |
| Golden Retriever | | Retriever | | RetrieverFlushingWater | 162 : 173 | 91 |
| Great Dane | | European Mastiff | | PinscherSchnauzer | 4 : 9 | 4 |
| Great Pyrenees | | Mediterranean | | PinscherSchnauzer | 2 : 3 | 0 |
| Greyhound | | UK Rural | | Sighthound | 6 : 8 | 0 |
| Griffon Nivernais | | NonClade | | Scenthound | 0 : 2 | 1 |
| Havanese | | Poodle | | CompanionToy | 7 : 10 | 2 |
| Hovawart | | NonClade | | PinscherSchnauzer | 1 : 2 | 1 |
| Icelandic Sheepdog | | Nordic Spitz | | SpitzPrimitive | 0 : 1 | 0 |
| Irish Setter | | Pointer Setter | | Pointing | 1 : 2 | 0 |
| Irish Terrier | | Terrier | | Terrier | 1 : 1 | 1 |
| Irish Wolfhound | | UK Rural | | Sighthound | 1 : 0 | 1 |
| Italian Greyhound | | UK Rural | | Sighthound | 1 : 0 | 1 |
| Jack Russell Terrier | | Terrier | | Terrier | 5 : 3 | 2 |
| Jagdterrier | | NonClade | | Terrier | 0 : 1 | 0 |
| Japanese Chin | | Asian Toy | | CompanionToy | 1 : 0 | 0 |
| Japanese Spitz | | NonClade | | SpitzPrimitive | 1 : 1 | 1 |
| Keeshond | | Nordic Spitz | | SpitzPrimitive | 0 : 3 | 0 |
| Kishu Ken | | NonClade | | SpitzPrimitive | 1 : 0 | 0 |
| Komondor | | Mediterranean | | SheepdogCattledog | 1 : 0 | 1 |
| Kooikerhondji | | NonClade | | RetrieverFlushingWater | 0 : 1 | 1 |
| Labrador Retriever | | Retriever | | RetrieverFlushingWater | 65 : 61 | 25 |
| Lagotto Romagnolo | | NonClade | | RetrieverFlushingWater | 0 : 1 | 1 |
| Leonberger | | Mediterranean | | PinscherSchnauzer | 1 : 0 | 0 |
| Lobito Herreno | | NonClade | | NonFCIBreed | 2 : 1 | 2 |
| Magyar agar (Hungarian Greyhound) | | NonClade | | Sighthound | 0 : 1 | 0 |
| Maltese | | Poodle | | CompanionToy | 1 : 6 | 0 |
| Manchester Terrier (Standard/Toy) | | NonClade | | Terrier | 0 : 1 | 0 |
| Markiesje | | NonClade | | NonFCIBreed | 0 : 1 | 0 |
| Mastiff | | NonClade | | PinscherSchnauzer | 2 : 2 | 1 |
| Miniature American Shepherd | | NonClade | | SheepdogCattledog | 4 : 4 | 4 |
| Miniature Pinscher | | Pinscher | | PinscherSchnauzer | 2 : 1 | 1 |
| Mixed breed | | NonClade | | MixedBreed | 1025 : 1119 | 778 |
| Munsterlander (Large/Small) | | Pointer Setter | | Pointing | 0 : 2 | 0 |
| Neapolitan Mastiff | | European Mastiff | | PinscherSchnauzer | 0 : 1 | 0 |
| Newfoundland | | Retriever | | PinscherSchnauzer | 10 : 6 | 2 |
| Norfolk Terrier | | Terrier | | Terrier | 0 : 1 | 1 |
| Norwegian Elkhound | | Nordic Spitz | | SpitzPrimitive | 0 : 1 | 0 |
| Norwich Terrier | | Terrier | | Terrier | 0 : 1 | 0 |
| Nova Scotia Duck-Tolling Retriever | | Retriever | | RetrieverFlushingWater | 3 : 1 | 0 |
| Olde English Bulldogge | | UK Rural | | NonFCIBreed | 0 : 1 | 0 |
| Owczarek Podhalanski (Polish Mountain Sheepdog) | | NonClade | | SheepdogCattledog | 1 : 0 | 0 |
| Papillon | | Toy Spitz | | CompanionToy | 2 : 3 | 1 |
| Parson Russell Terrier | | Terrier | | Terrier | 0 : 3 | 1 |
| Pekingese | | Asian Toy | | CompanionToy | 1 : 1 | 0 |
| Pembroke Welsh Corgi | | UK Rural | | SheepdogCattledog | 12 : 18 | 9 |
| Perro de Presa Canario | | NonClade | | PinscherSchnauzer | 1 : 0 | 0 |
| Plott | | NonClade | | NonFCIBreed | 1 : 0 | 0 |
| Pointer | | NonClade | | Pointing | 3 : 0 | 0 |
| Pomeranian | | Small Spitz | | SpitzPrimitive | 2 : 8 | 4 |
| Poodle (Standard, Medium, Miniature, Toy) | | Poodle | | CompanionToy | 29 : 32 | 15 |
| Portuguese Podengo | | NonClade | | SpitzPrimitive | 1 : 0 | 0 |
| Portuguese Podengo Pequeno | | NonClade | | SpitzPrimitive | 1 : 0 | 0 |
| Portuguese Sheepdog | | NonClade | | SheepdogCattledog | 0 : 1 | 0 |
| Portuguese Water Dog | | Poodle | | RetrieverFlushingWater | 6 : 2 | 1 |
| Pug | | Toy Spitz | | CompanionToy | 1 : 8 | 1 |
| Puli | | Hungarian | | SheepdogCattledog | 2 : 0 | 1 |
| Pyrenean Shepherd | | NonClade | | SheepdogCattledog | 1 : 0 | 1 |
| Rat Terrier | | American Terrier | | NonFCIBreed | 2 : 1 | 1 |
| Redbone Coonhound | | Scent Hound | | NonFCIBreed | 0 : 1 | 0 |
| Rhodesian Ridgeback | | European Mastiff | | Scenthound | 7 : 2 | 4 |
| Rottweiler | | Drover | | PinscherSchnauzer | 6 : 8 | 6 |
| Russell Terrier | | NonClade | | Terrier | 0 : 1 | 0 |
| Saluki | | Mediterranean | | Sighthound | 1 : 0 | 0 |
| Samoyed | | NonClade | | SpitzPrimitive | 6 : 8 | 6 |
| Sarplaninac | | NonClade | | PinscherSchnauzer | 0 : 1 | 1 |
| Schapendoes | | NonClade | | SheepdogCattledog | 1 : 0 | 0 |
| Schipperke | | Toy Spitz | | SheepdogCattledog | 0 : 1 | 0 |
| Schnauzer (Giant/Standard/Miniature) | | Schnauzer | | PinscherSchnauzer | 6 : 8 | 6 |
| Scottish Terrier | | Terrier | | Terrier | 0 : 1 | 0 |
| Shetland Sheepdog | | UK Rural | | SheepdogCattledog | 6 : 3 | 1 |
| Shiba Inu | | Asian Spitz | | SpitzPrimitive | 4 : 4 | 5 |
| Shih Tzu | | Asian Toy | | CompanionToy | 8 : 12 | 2 |
| Siberian Husky | | Asian Spitz | | SpitzPrimitive | 11 : 11 | 6 |
| Silken Windhound | | NonClade | | NonFCIBreed | 0 : 4 | 3 |
| Silken Windsprite | | NonClade | | NonFCIBreed | 0 : 1 | 0 |
| Silky Terrier | | Terrier | | Terrier | 2 : 0 | 0 |
| Soft-Coated Wheaten Terrier | | Terrier | | Terrier | 3 : 5 | 1 |
| Spanish Greyhound (Galgo Espanol) | | NonClade | | Sighthound | 1 : 3 | 2 |
| Spanish Water Dog | | NonClade | | RetrieverFlushingWater | 0 : 1 | 0 |
| Spinone Italiano | | Pointer Setter | | Pointing | 0 : 2 | 0 |
| St. Bernard | | Alpine | | PinscherSchnauzer | 1 : 1 | 0 |
| Stabyhoun | | NonClade | | Pointing | 1 : 0 | 1 |
| Staffordshire Bull Terrier | | European Mastiff | | Terrier | 2 : 4 | 2 |
| Swedish Vallhund | | Nordic Spitz | | SpitzPrimitive | 1 : 0 | 0 |
| Teddy Roosevelt Terrier | | NonClade | | NonFCIBreed | 2 : 2 | 2 |
| Thai Ridgeback | | NonClade | | SpitzPrimitive | 0 : 1 | 0 |
| Tibetan Mastiff | | Asian Spitz | | PinscherSchnauzer | 1 : 2 | 1 |
| Tibetan Terrier | | NonClade | | CompanionToy | 1 : 1 | 0 |
| Treeing Walker Coonhound | | NonClade | | NonFCIBreed | 0 : 1 | 0 |
| Vizsla | | Pointer Setter | | Pointing | 4 : 3 | 2 |
| Weimaraner | | Pointer Setter | | Pointing | 1 : 4 | 1 |
| Welsh Springer Spaniel | | NonClade | | RetrieverFlushingWater | 1 : 0 | 0 |
| Welsh Terrier | | NonClade | | Terrier | 3 : 0 | 0 |
| West Highland White Terrier | | Terrier | | Terrier | 5 : 7 | 3 |
| Whippet | | UK Rural | | Sighthound | 4 : 0 | 0 |
| Wirehaired Pointing Griffon | | Pointer Setter | | Pointing | 1 : 0 | 0 |
| Xoloitzcuintli (Standard/Miniature/Toy) | | New World | | SpitzPrimitive | 0 : 1 | 1 |
| Yakutian Laika | | NonClade | | SpitzPrimitive | 1 : 0 | 0 |
| Yorkshire Terrier | | Terrier | | Terrier | 6 : 8 | 2 |

Table S3. Distribution of total sample of dogs across genetic clades, in alphabetical order.

| **Clade** | **N** | **% Total Sample** |
| --- | --- | --- |
| Alpine | 15 | 0.33% |
| American Terrier | 22 | 0.49% |
| American Toy | 25 | 0.56% |
| Asian Spitz | 195 | 4.34% |
| Asian Toy | 23 | 0.51% |
| Continental Herder | 51 | 1.13% |
| Drover | 29 | 0.64% |
| European Mastiff | 80 | 1.78% |
| Hungarian | 2 | 0.04% |
| Mediterranean | 56 | 1.25% |
| New World | 100 | 2.22% |
| Nordic Spitz | 32 | 0.71% |
| Pinscher | 3 | 0.07% |
| Pointer Setter | 46 | 1.02% |
| Poodle | 128 | 2.85% |
| Retriever | 483 | 10.74% |
| Scent Hound | 85 | 1.89% |
| Schnauzer | 14 | 0.31% |
| Small Spitz | 10 | 0.22% |
| Spaniel | 44 | 0.98% |
| Terrier | 109 | 2.42% |
| Toy Spitz | 19 | 0.42% |
| UK Rural | 259 | 5.76% |

**Supporting References**

*FCI Breeds Nomenclature*. Retrieved September 3, 2024, from https://fci.be/en/Nomenclature/

Parker, H. G., Dreger, D. L., Rimbault, M., Davis, B. W., Mullen, A. B., Carpintero-Ramirez, G., & Ostrander, E. A. (2017). Genomic Analyses Reveal the Influence of Geographic Origin, Migration, and Hybridization on Modern Dog Breed Development. *Cell Reports*, *19*(4), 697–708. https://doi.org/10.1016/j.celrep.2017.03.079

Rosseel, Y. (2012). lavaan: An R package for structural equation modeling. *Journal of statistical software*, *48*, 1-36. <https://10.18637/jss.v048.i02>
